# A Crowdsensing Based Analytical Framework for Perceptional Degradation of OTT Web Browsing

**DOI:** 10.3390/s18051566

**Published:** 2018-05-15

**Authors:** Ke Li, Hai Wang, Xiaolong Xu, Yu Du, Yuansheng Liu, M. Omair Ahmad

**Affiliations:** 1 College of Smart City, Beijing Union University, Beijing 100101, China; whyxdt@163.com (H.W.); 151081210202@buu.edu.cn (X.X.); 2 College of Robotics, Beijing Union University, Beijing 100101, China; duyu@buu.edu.cn (Y.D.); yuansheng@buu.edu.cn (Y.L.); 3 Department of Electrical and Computer Engineering, Concordia University, Montreal, QC H3G IM8, Canada; omair@ece.concordia.ca

**Keywords:** service perception, mobile crowdsensing, network measurement, over-the-top service

## Abstract

Service perception analysis is crucial for understanding both user experiences and network quality as well as for maintaining and optimizing of mobile networks. Given the rapid development of mobile Internet and over-the-top (OTT) services, the conventional network-centric mode of network operation and maintenance is no longer effective. Therefore, developing an approach to evaluate and optimizing users’ service perceptions has become increasingly important. Meanwhile, the development of a new sensing paradigm, mobile crowdsensing (MCS), makes it possible to evaluate and analyze the user’s OTT service perception from end-user’s point of view other than from the network side. In this paper, the key factors that impact users’ end-to-end OTT web browsing service perception are analyzed by monitoring crowdsourced user perceptions. The intrinsic relationships among the key factors and the interactions between key quality indicators (KQI) are evaluated from several perspectives. Moreover, an analytical framework of perceptional degradation and a detailed algorithm are proposed whose goal is to identify the major factors that impact the perceptional degradation of web browsing service as well as their significance of contribution. Finally, a case study is presented to show the effectiveness of the proposed method using a dataset crowdsensed from a large number of smartphone users in a real mobile network. The proposed analytical framework forms a valuable solution for mobile network maintenance and optimization and can help improve web browsing service perception and network quality.

## 1. Introduction

Internet services are usually characterized by low real-time capability and reliability but high flexibility. In contrast, services over a telecom network are typically highly stable and reliable—a point of pride for telecom operators. To reinforce their competitive advantages, telecom operators expend considerable efforts to maintain and improve network quality in their so-called “network-centric” operation and maintenance modes.

However, one problem that arises in a network-centric operating context is that the operators usually address the service problems solely from the perspective of network quality. They ignore the end-user experience. However, given the rapid growth in mobile networks and smartphones, Over-The-Top (OTT) services have experienced rapid development, becoming a major part of mobile network carrier services and replacing conventional services. This change is notably exemplified by WeChat’s domination of conventional voice and short message service. Mobile networks are threatened with becoming pure pipelines. Simply ensuring network quality is no longer the sole guarantor of service experience. Therefore, to ensure the user experience and reduce customer turnover, it has become crucial to revolutionize the methodology by which mobile network quality is tested, evaluated and optimized.

Conventional mobile network optimization (MNO) relies primarily on methods such as drive tests (DT) and call quality tests (CQT), and optimization techniques employ key performance indicators (KPI) as metrics for evaluating network quality. However, this approach focuses on evaluating and optimizing networks from the perspective of the network operators rather than from a service perspective. Consequently, there is an increasingly serious disconnect between network quality and service experience in the mobile Internet scenario. Thus, quality of experience (QoE) has garnered increasing attention from both industry and academia. Researchers have conducted extensive studies to acquire data to construct key quality indicator (KQI) or Quality of Service (QoS) metrics and improve the methodology for evaluating QoE [1,2,3,4,5,6,7,8].

Although user perceptions of networks have become increasingly crucial, the methods for QoE data acquisition and evaluation are still limited. Most of these methods involve probing the network, for example, deep packet inspection (DPI) [9]. Generally, however, measurements made at closer proximity to end-users provide a more accurate picture of service perception; consequently, it is crucial to obtain information from the user end. Some operators have begun to employ big data driven methods to supply more accurate evaluations of end-users’ perceptions concerning network and service quality [10]. It is believed that this approach can improve cooperation among relevant operator sectors and thus improve the service perceptions. Furthermore, the data can also be utilized to provide more personalized marketing and customer services. This method of data collection is referred to as crowdsourcing-based user perception (CUP).

The ultimate goal of service perception is to guide network optimization to improve users’ perceptions. To achieve this, one must first analyze and locate the factors that affect service perception. Taking OTT web browsing services as the main target, this article investigates the user perception problem discussed above based on large amounts of perceptual data collected from end-users’ smartphones in live networks. Multiple data analytics and statistical methods are employed to systematically analyze the factors that affect user’s service perceptions.

In addition, this article provides a unified framework for identifying and analyzing the factors that affect service perception degradation and the significance of their contributions.

The remainder of this paper is organized as follows: Section 2 presents related work. In Section 3, the factors that impact OTT service perception are analyzed, together with an analysis of their relationships and how these factors affect the KQIs. Section 4 describes a comprehensive analytical framework and a detailed algorithm for locating the root causes of service perception degradation with crowdsensed data. In Section 5, a case study is presented to validate the proposed framework by utilizing the perceptional data crowdsensed from a provincial Long Term Evolution (LTE) network in China. Finally, Section 6 concludes the paper and discusses future work.

## 2. Related Work

This section provides an overview and comparison of typical network measurement and evaluation methods and presents the methodology for the CUP data acquisition and evaluation techniques employed in this article.

### 2.1. Network Measurement and Evaluation

Generally, network and service measurements are performed in two ways: active testing and passive monitoring. Active testing, represented by DT and CQT, is the most conventional method. For example, ref. [11] employs active testing initiated from the terminal side. Active testing is transparent and fully controlled, but its main disadvantage is the limited number of samples due to cost control. It is only simulations of actual user behavior in limited time and places.

Because of the intrinsic big data nature of data generated in mobile networks through passive monitoring, how to best utilize the huge amount of data to advance operators’ business is a field with increasing attention. Ref. [12] presents current status of network-side data acquisition and analysis. Of which DPI is the most widespread passive monitoring on the network side. DPI monitors all the subscribers in the network. Nevertheless, deploying and operating DPI involves high costs, and it is nearly impossible to benchmark across the operators.

**Figure 1:**
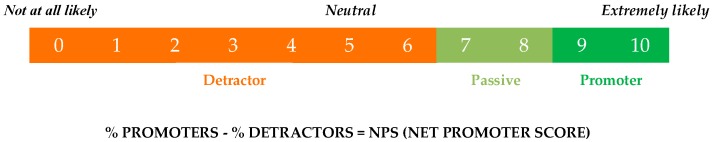
Standard for net promoter score (NPS) evaluation.

Despite the above-mentioned objective measurements, subjective measurements, such as net promoter score (NPS) [13,14] can be conducted to obtain end-users’ perceptions of service quality. As a popular metric to analyze customer loyalty in the business field, it has recently become the favor of operators. It measures the likelihood that a customer will recommend services to others. The most widely employed NPS data collection method is survey questionnaires. Figure 1 provides the general standard for evaluating NPS.

Unfortunately, subjective NPS scores are highly influenced by the survey process, the sampling time and volume, customers’ mental states, and even promotional gifts. Consequently, it may result in data with low validity.

Accompanying the increasing popularity of smartphones and portable devices carrying various sensors, crowdsourcing-based measurements at the terminal side have gained traction in academia and industrial sectors. Ref. [15] denotes this new sensing paradigm as Mobile Crowdsensing (MCS) and divides MCS into two types: Participatory Sensing and Opportunistic Sensing. The former requires individuals to actively participate by contributing sensing data (for example, uploading pictures or reporting real-time traffic), while the latter uses passive, autonomous methods that usually do not require user’s participation.

In recent years, many efforts have been made to improve specific techniques for MCS. Many of them focus on the generic framework of the MCS system [16,17,18,19]. For example, in [18] a distributed framework has been proposed for gathering information in cloud-based mobile crowd sensing systems with opportunistic reporting. Ref. [19] proposed a so-called Piggyback Crowdsensing system for collecting mobile sensor data from smartphones that lowers the energy overhead of user participation, by exploiting smartphone application (APP) opportunities.

Some researches deal with challenges that MCS is facing [20,21,22,23]. Ref. [20] investigated the opportunistic characteristics of human mobility from the perspectives of both sensing and transmission. A user recruitment policy for data acquisition in mobile crowdsensing systems has been proposed in [21], to minimize the cost and maximize the return. In [23], the authors acquired service perception data through both active measurements in the lab and passive smartphone monitoring of participating users and used the collected data to evaluate the QoE performance of common apps such as YouTube, Facebook, Chrome, and Google Maps.

CUP, the method discussed in this article, uses the opportunistic sensing of MCS approach. It focuses primarily on how to collect users’ service experience data from smart devices through monitoring and subsequent analysis service quality and evaluation. Because CUP is based on big data generated by monitoring real service experiences, it is naturally superior in its objectivity, accuracy, integrity, and time scope. Thus, it provides a holistic reflection of real condition of the networks and services. Its greatest challenge lies in placing the data acquisition agent onto users’ smartphones. This agent can be implemented as a stand-alone app but is more commonly distributed as a software development kit (SDK) plug-in bundled with other apps. Another challenge is how to avoid invading users’ privacy. In addition, since data collection normally operates at the operating system (OS) level, the wireless parameters it acquires are relatively limited. Network data such as air interface signaling, which are widely used for network optimization, are impossible to obtain.

### 2.2. Crowdsourcing-Based User Perception

**Figure 2:**
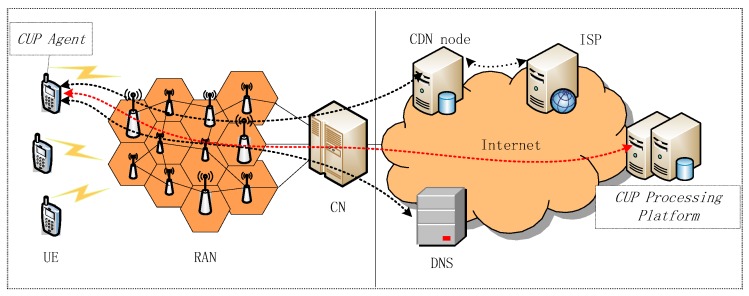
Architecture of a crowdsourcing-based user perception (CUP)-based system.

In general, a client-server architecture is employed for the CUP system, as illustrated in Figure 2.

The system generally consists of two parts: the data acquisition agent, and a data processing platform. The data acquisition agent is pre-installed in a large number of user equipment (UE) and runs in the background to monitor users’ OTT service behaviors. In this article, we select web browsing as the target service for the following analysis. Under certain conditions (e.g., the user launches a web browser APP to visit the website of a target Internet service provider (ISP)), the agent is then triggered to collect the service perception information. The information is transferred to the data processing platform on the cloud side periodically through radio access network (RAN), core network (CN) and Internet, as indicated in Figure 2 with red dotted line.

The uploaded raw data are pre-processed by the data-processing platform, which includes removal of invalid data, sensitive personal information, and so on. To protect the privacy of the mobile subscribers to the highest extent, confidential information such as phone numbers and the body text of SMS messages is not collected. Moreover, only the terminal and subscriber ID (i.e., international mobile equipment identity (IMEI) and international mobile subscriber identity (IMSI)) are collected for user identification, and the data are permuted and encrypted before use. The CUP processing platform also provides the system user with a friendly interface through which to view the collected data and analytical results. The parameters of data acquisition and reporting, including list of target websites, reporting period, etc. are configurable in the platform.

Data collected by the agents are grouped into 3 categories.

1. Service Perceptional KQI

This is the most important part of the whole dataset. For different types of OTT services (i.e., web browsing, video, instant messaging, etc.), a class of representative KQI indices is defined based on the unique properties and users’ perceptions of the service.

To our knowledge, the most representative perceptional indices of OTT web browsing are time delays at each stage of the entire hypertext transfer protocol (HTTP) process. Specifically, ref. [10] defines metrics includes first packet delay and page delay. This article employs the following indices, and they are identified by tracking and analyzing the real-time logs of target web browsing APP.

(1) First Packet Delay (*D_k_*): The time between a user triggering a webpage request and the handset receiving the first HTTP 200 OK packet from the target server, calculated as follows:

(1) $$D_{k}=T_{200}-T_{req},$$

where *T_req_* denotes the timestamp when the webpage request was triggered, and *T*_200_ denotes the timestamp when the first HTTP 200 OK packet arrives at the handset.

**Figure 3:**
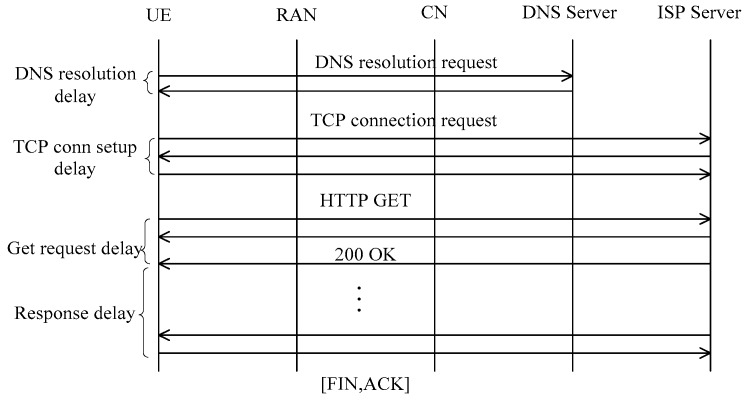
Process of over-the-top (OTT) web browsing over hypertext transfer protocol (HTTP).

Generally, an HTTP webpage browsing process consists of several stages (i.e., domain name system (DNS) resolution, transmission control protocol (TCP) connection setup, and HTTP interaction) as illustrated in Figure 3. Therefore, the first packet delay can be further divided into 3 segments:

(2) $$D_{k}=D_{dns}+D_{tcp}+D_{get,}$$

where

(3) $$\{\begin{array}{c} D_{dns}=T_{dns}-T_{req}\\ D_{tcp}=T_{tcp}-T_{dns}\\ D_{get}=T_{200}-T_{tcp} \end{array},\;$$

in which:

(a) *D_dns_* refers to DNS resolution delay, which is the elapsed time from the webpage request until the handset receives the DNS resolution result (i.e., *T_dns_*). It should be noted, however, that the air interface setup delay is also included in *D_dns_* if there is no air interface connection setup at the time of webpage request.
(b) *D_tcp_* is the TCP connection setup delay, referring to the time between the completion of DNS resolution and the TCP confirmation being sent by the handset (i.e., *T_tcp_*).
(c) *D_get_* is the GET request delay, which is the elapsed time between the handset’s TCP confirmation and the arrival of the first TCP packet at the handset.

(2) Page Delay (*D_p_*): This delay refers to the time between the triggering of the webpage request and the time at which the full HTML page content (excluding the transferring and loading of non-text resources) is received and rendered by the browser APP, which is calculated by:

(4) $$D_{p}=D_{k}+D_{res},$$

where *D_res_* is the response delay, i.e., the time between the receiving of the HTTP 200 OK packet and the sending of the [FIN, ACK] packet (i.e., *T_res_*), calculated as:

(5) $$D_{res}=T_{res}-T_{200}.$$

2. Wireless Environmental Information

Along with the service perception information, the agent also collects instantaneous wireless environment parameters via the generic APIs, including network type, signal strength, signal quality, cell ID, etc. They are primary indicators of the network quality at the time of data acquisition.

For LTE network, signal strength and quality are represented by reference signal received power (RSRP) and reference signal received quality (RSRQ), respectively, while the cell ID is a unique combination of three parameters, i.e., {TAC, eNodeB ID, and cell ID}.

3. Terminal and Positioning Information

Terminal information includes information such as the user ID (i.e., IMSI), handset ID (i.e., IMEI), model type, OS version, etc. Positioning information includes current location (longitude and latitude), the positioning method and precision. In case the GPS module is enabled by the user, the GPS longitude and latitude are collected by the agent. Otherwise, the agent will acquire current location with the 3rd party network augmented positioning APIs like Baidu or Google positioning. The network augmented positioning is less accurate and consumes less battery than GPS positioning. The agent doesn’t trigger the GPS positioning initiatively, to avoid interference to the user and save battery energy.

Based on the acquired data samples of the abovementioned information, a perceptional evaluation of OTT service quality can be made through a variety of KQI/QoS-QoE models.

The mapping between KQI or QoS to end-user perception has been extensively researched in recent years. In [24], a generic exponential relationship between QoE and QoS was proposed through an exponential interdependency of QoE and QoS (namely, IQX hypothesis), and validated with experimental data for both streaming and web surfing services. In [25], the relationship between QoE and QoS for web browsing was investigated by probing the packets generated by the service through either dial-up or high-speed Internet connections. Then a quantitative non-linear relationship was achieved, which shows that bandwidth plays more crucial role than network latency on the level of user satisfaction. Ref. [26] presents a close look on how the waiting time impact on user's QoE for web-based services.

In industrial practice, however, the KQI/QoS-QoE mapping model employed is somewhat simple and straightforward. For instance, a hierarchical linear model called Ratio of Qualified Samples (RQS), was proposed in [10] and deployed by China Telecom in evaluating the quality of OTT services for its provincial divisions. Unlike QoE score which is often in the range of 1–5, RQS is a percentage ranging from 0 to 100%. The higher the RQS, the better the quality of service. From bottom to top, it consists of four levels of indices: KQI-level, service-level, network-level and overall RQS. Each index is a weighted sum of the lower-level indices. The RQS index tree is illustrated in Table 1.

In conclusion, we can see that OTT web browsing service significantly differentiates itself from the traditional web browsing in the following aspects.

Firstly, both the web page and web browser of OTT service are optimized to guarantee user perception and save some cost. As seen in Table 2 (taking two most popular websites in China as example), that the page size and number of resources of the homepage is much fewer than that of traditional ones. The downloading mechanism of web browser APPs is generally on-demand—initially only the resources of the first screen of the page is downloaded and rendered, and the remaining will be downloaded in case the user scrolls down. Secondly, the definition of KQI indices of OTT web browsing considers mobile-specific issues, while traditional web service does not. For instance, the DNS delay consists air interface delay. Furthermore, the impact factors of user perception for both types of service is quite different. In contrast to conventional web browsing running on computers, an OTT web browsing involves the whole mobile network entities including UE, RAN and CN during the process (i.e., the left half of Figure 2). The end-user’s service perception is much more vulnerable to the fast-changing radio environment, which is not the case for conventional service.

## 3. Impact Factors of Perceptional Degradation of OTT Services

In this section, the factors that possibly impact on end-user’s OTT service perception are presented and compared. Then taking quality of access network coverage, system load, and ISP webpage as examples, we give more detailed analysis on the relationship between the impact factors and perceptional KQIs of OTT web browsing. The relationship between KQI indices is also identified.

### 3.1. Impact Factors of OTT Service Perception

As illustrated in Figure 2, for an OTT web browsing service, the end-to-end service perception is subject to factors that cover all the parties involved in the service process. Obviously, it is much more complicated than that of conventional web browsing. Generally, the factors that impact an OTT service includes the following 6 dimensions: radio access network, core network, temporal domain, terminal, user, and ISP.

(1) Access Network: Considering the large temporal and spatial variation of the wireless propagation environment, this factor is thought to have the most influence on service perceptions—it is not only significant but also unstable. More specifically, a high-quality wireless signal means qualified coverage (i.e., the signal in the area is sufficiently strong and experiences little interference). As the malfunction of one base station in the access network affects only the users within its coverage, range of influence of RAN is medium.
(2) Core Network (Including CN Equipment and the Physical Links Among Them): As the highest-level equipment in the whole mobile network, CN is crucial to the overall network performance; thus, it is always located in a fully controlled server room and maintained with high attention. Thus, it rarely malfunctions and therefore has little impact on overall service perception.
(3) Temporal Domain (System Load): Service attempts are temporally random; therefore, system load is also temporally random. Temporal differences in service demand intensity inevitably impact the wireless network load, the core network, the ISP website, and, finally, the service perception. Higher system loads generally result in lower service perceptions.
(4) Terminal: One significant characteristic of smartphones, especially Android phones, is fragmentation among many brands and models. Different key component configurations, such as CPU and memory, lead to different hardware performances and, thus, different service perceptions. Its range of influence is medium to large. For best-selling terminals, low service perception resulting from the design or production defect would impact a large number of users.
(5) User: The service perception of a specific user is generally different from that of others, even under the same circumstances. These differences may result from the user’s personal phone-use habits, the hardware and software implementations of that user’s specific phone or the user’s psychological anticipation of satisfactory service usage.
(6) ISP: Almost all the OTT service providers employ content distribution network (CDN) [27] technology to provide content service as close to end-users as possible. Therefore, service perceptions of the same ISP website can differ substantially among different regions due to location, processing capability and bandwidth variations in the CDN servers responsible for those regions. Malfunction of ISP server would influence a large number of users visiting the website, while malfunction of one CDN nodes would influence less.

Additionally, for HTTP-based services such as web surfing, a DNS server is required to provide domain name translation services. The DNS server may be placed inside the operator’s network or implemented through a 3rd-party server pool. The performance and bandwidth of DNS servers also affect user perceptions.

In conclusion, Table 3 presents a subjective view of the abovementioned impact factors and their characteristics.

To further consolidate our views given above, hereafter we analyze and verify the substantial influences of the impact factors on users’ perceptions by utilizing the large volume of OTT web browsing service perception data crowdsourced from the live LTE network during July and August of 2017. The data acquisition agent SDK was embedded in the online customer service APP of local operator we are cooperating with, and the target users of the APP are its subscribers. The data are acquired from five representative provinces throughout China, i.e., Beijing, Jiangsu, Guangdong, Sichuan, and Shannxi. Total size of the dataset is 7,434,030. The target websites here are the nine most popular websites in China: Sina, Baidu, Sohu, Taobao, People, iFeng, Tencent, Weibo, and Netease.

### 3.2. Impact of Quality of Coverage on Service Perception

For wireless access networks, good signal coverage is an important factor in service perception. The most basic index is the signal strength at the service location, followed by the signal quality, that is, the interference level. In LTE networks, these are the RSRP and RSRQ, respectively. In general, better signal strength and signal quality lead to higher successful data transmission rates and lower delays in air interface and, thus, better service experiences.

We first analyze the impact of coverage capability on service perception metrics, that is, the correlation between signal strength and signal quality and service perception metrics.

**Figure 4:**
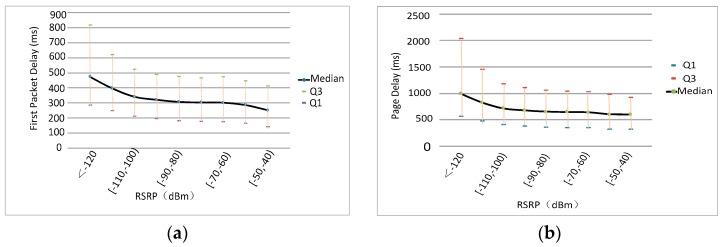
Distribution of first packet delay and page delay vs. RSRP. (**a**) RSRP vs. First Packet Delay; (**b**) RSRP vs. Page Delay.

The distribution of average first packet delay and page delay under different RSRP levels is shown in Figure 4.

Here, Q1 and Q3 refer to the 1/4 and 3/4 quantiles of the KQI index in each RSRP segment, respectively. When RSRP is less than −100 dBm (for LTE networks, we generally define weak coverage as an RSRP below −110 dBm), both KQIs deteriorate significantly. This shows that service perception is highly sensitive to the network coverage quality. The analysis for RSRQ vs. first packet delay and page delay results in the same conclusion.

Therefore, the quality of wireless environment is the key factor that operators should consider to maintain and optimize the service perception.

### 3.3. Impact of Service Intensity on Service Perception

**Figure 5:**
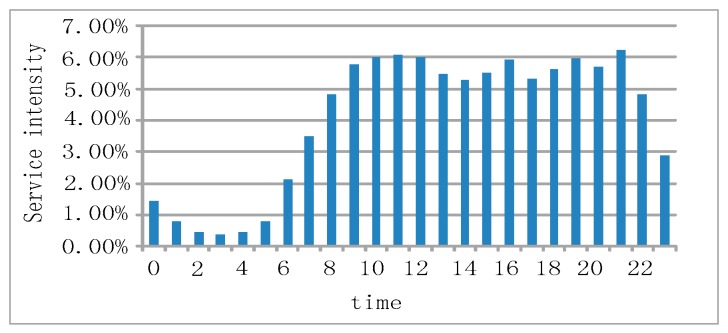
Service intensity of web browsing at different hours of the day.

We know that the transmission capacity of the network varies under different network loads. However, because it is difficult to obtain network load information from the terminal end, we instead analyze the correlation between service perception and service intensity during different hours of the day. Generally, service intensity varies at different times of the day. Figure 5 shows the percentage of web browsing service attempts for each hour of the day. Between 23:00 and 7:00 of the next day, service intensity is low, which means that the network load is low as well.

**Figure 6:**
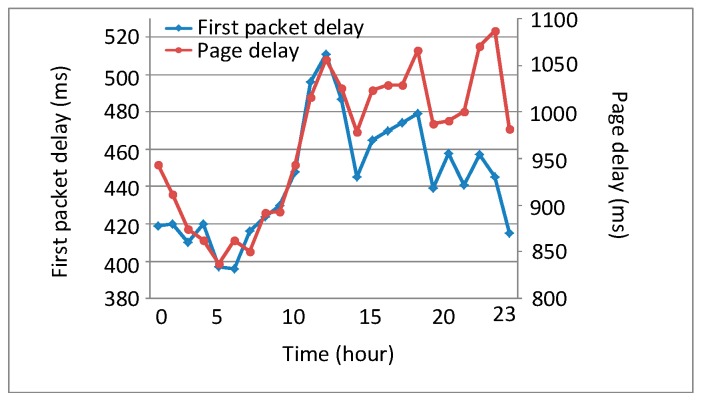
First packet delay and page delay at each hour of the day.

The statistics of service perception KQIs at each hour of the day are illustrated in Figure 6 and show that the fluctuation of perceptional indices with respect to time is highly similar to that of the service intensity. This shows that service intensity (and thus network load) also has a significant impact on service perceptions.

Further, we can perform a quantitative evaluation of the correlation between these factors. The conventional method to quantitatively evaluate correlation is Pearson correlation coefficient (PCC), which utilizes covariance and standard deviation estimates. The PCC of two groups of samples can be computed by the following equation:

(6) $$r=\frac{\sum_{i=1}^{n}\left(X_{i}-\bar{X}\right)\left(Y_{i}-\bar{Y}\right)}{\sqrt{\sum_{i=1}^{n}{\left(X_{i}-\bar{X}\right)}^{2}}\sqrt{\sum_{i=1}^{n}{\left(Y_{i}-\bar{Y}\right)}^{2}}},$$

where {*X_i_*, *i* = 1~*n*}{*Y_i_*, *i* = 1~*n*} are the two groups of observed samples, and $\bar{X}$ and $\bar{Y}$ are their mathematical expectations, respectively.

To further validate this observation, the maximum information coefficient (MIC) approach is also employed in this paper [28,29]. The MIC method has been proved to be capable of detecting the association of various functions or non-functions extensively, which is superior to other methods, such as PCC, the Kraskov mutual information estimator [30], and the Spellman estimator [31]. It is shown in [29] that in the case of a probability approaching 1 as the sample size grows, the MIC score approaches 1 for all never-constant noiseless functional relationships or a larger class of noiseless relationships. And it approaches 0 for statistically independent variables.

Below is the definition of MIC and the characteristic matrix [29]. Given a finite set $D\subset {\mathbb{R}}^{2}$ of ordered pairs, the *x*-values and *y*-values of *D* are partitioned into *x* and *y* bins, respectively, allowing empty bins. We call such a pair of partitions an *x*-by-*y* grid. Given a grid *G*, let *D*|*_G_* be the distribution induced by the points in *D* on the cells of *G*. For a fixed *D*, different grids *G* result in different distributions *D*|*_G_*.

Then for the positive integers *x*, *y*, we define

(7) $$I^{*}\left(D,x,y\right)=\max I\left(D{|}_{G}\right),$$

where max(•) is over all the grids *G* with *x* columns and *y* rows, and *I*(*D*|*_G_*) denotes the mutual information of *D*|*_G_*.

Suppose the characteristic matrix M(D) of the finite set *D* is given by:

(8) $$M{\left(D\right)}_{x,y}=\frac{I^{*}\left(D,x,y\right)}{\log\;\min\left\{x,y\right\}}.$$

Then the MIC of the dataset D with sample size *n* and grid size less than *B*(*n*) is defined as:

(9) $$\mathrm{MIC}\left(D\right)=\underset{xy<B\left(n\right)}{\max}\left\{M{\left(D\right)}_{x,y}\right\},$$

where $\omega \left(1\right)<B\left(n\right)\leq O\left(n^{1-\varepsilon }\right)\;\mathrm{for}\;\mathrm{some}\;0<\varepsilon <1.$ *B*(*n*) is suggested to be *n*^0.6^.

**Figure 7:**
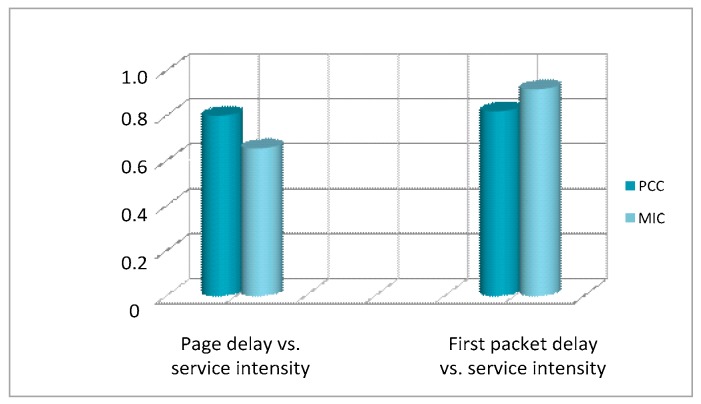
Quantitative analysis of key quality indicator (KQI) indices vs. service intensity.

Utilizing the PCC and MIC methods, we calculated and compared the correlation between the first packet delay/page delay and the service intensity at each hour of the day, as shown in Figure 7.

Obviously, both the first packet delay and the page delay show significant correlations with the service intensity and network load.

### 3.4. Relationship of ISP Webpage and Service Perception

**Figure 8:**
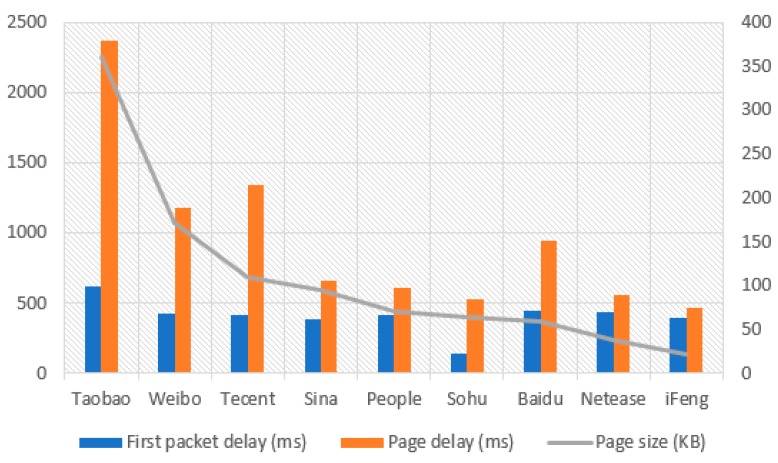
KQI of each website.

The page sizes of different websites are generally different. Therefore, the page delay may be affected by the page size. Therefore, the statistics of the service perception indices for each website are shown in Figure 8.

As Figure 8 shows, no significant difference exists among the first packet delays of different websites. This is because the first packet delay depends mainly on the response speed of the website to the webpage request from the terminal side. In this situation, page size is irrelevant to first packet delay. The only exception is Sohu whose first packet delay is only 142 ms, and the reason is still unknown. Conversely, the page delay metric is highly correlated with the page size. In addition, the number of page requests for each website in a certain area changes constantly. Therefore, when defining a disqualification threshold for page delay, it is preferable to consider differences in both the page size of each target website and the page visit intensity of these websites. In Section 4.2 a detailed suggestion for solving this problem is given.

### 3.5. Relationships among KQI Indices

According to Equation (4), page delay is the inclusion of first packet delay. Thus, it is interesting to investigate the quantitative relationship between the two indices. The PCC and MIC calculation results are 0.78 and 0.82, respectively. Therefore, apparently they are highly related with each other.

## 4. Design of the Analytical Framework for Service Perception Degradation

In this section, firstly an analytical framework for service perception degradation is designed and the detailed algorithm is presented. Then how to determine the key parameters of the proposed algorithm is addressed.

### 4.1. Design of Analytical Framework

From the above discussion, the end-to-end service perception is influenced by several factors, and many functional parties, both inside and outside the operator, are involved in guaranteeing end-to-end perception. For instance, the network optimization and network management departments are responsible for wireless and core network problems, respectively, while most website problems can be solved only by the ISP and CDN vendors. In many cases, close cooperation is necessary to solve perceptual problems. Therefore, how to best utilize mobile big data in discovering the root causes of perceptional degradation remains a significant challenge.

Generally, this problem can be approached in a top-down manner. First, we should analyze and locate the problems at the network level and then narrow them down to the cell level to locate the cause of degradation in a disqualified cell. The necessity of cell-level analysis is that all state-of-the-art mobile network optimization is carried out at the cell granularity (e.g., adjusting the cellular site height, downtilt and azimuth of the antenna, and the engineering parameters of the cell such as its max transmission power and handover threshold).

In the following, a network-level analytical framework for the degradation of OTT web browsing services is designed. The overall framework for other OTT services such as video and IM could be designed similarly, with adequate adaptations.

**Figure 9:**
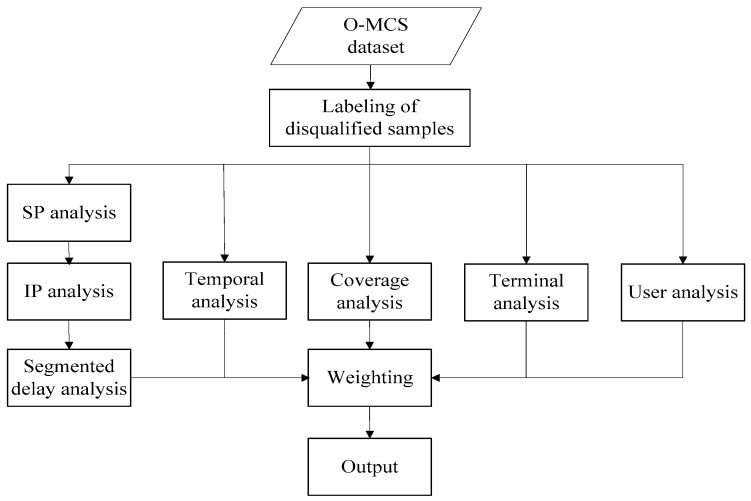
Analytical framework of service perception degradation.

From the above analysis, we know that the impact factors of web browsing service perception at the network level consists of the ISP website (including the IP pool and CDN nodes), DNS server, network load, segmented delay, signal strength and quality in geographical areas, the terminal, and the user. By considering these factors, the overall framework can be designed as follows (Figure 9).

Taking the OTT web browsing service in the LTE network as an example, the input dataset is denoted as $D=\left\{(x_{i},Y_{i})|1\leq i\leq m\right\}$, where $m=\overset{N}{\underset{i=1}{\sum }}m_{i}$, $m_{i}$ refers to the number of samples of the *i*-th website, *N* is the total number of websites, and all the entries of ***Y*** are null initially. Here, $x=\left\{x_{1},x_{2},\ldots ,x_{d}|d=18\right\}$ denotes the attributes of an instance, in which the 18 attributes are {*D_k_*, *D_p_*, *D_dns_*, *D_tcp_*, *D_get_*, *D_res_*, Rx, RSRQ, date, time, user ID, phone model, TAC, eNodeBID, cell ID, ISP, CDN IP, DNS IP} in that sequence. The attributes {*D_k_*, *D_p_*, *D_dns_*, *D_tcp_*, *D_get_*, *D_res_*, Rx, RSRQ, date, time} are numerical data, while the remaining attributes are nominal data.

Denote $Y=\left\{y_{1},y_{2},\ldots ,y_{q}|q=8\right\}$ as the binary label set of dataset ***D***, corresponding to the attributes {*D_k_*, *D_p_*, *D_dns_*, *D_tcp_*, *D_get_*, *D_res_*, RSRP, RSRQ}, respectively.

Firstly, the disqualified samples in the dataset need to be labelled with the predefined disqualification thresholds {*T_j_*, *j* = 1~8}. For each instance of ***D***, the labels are determined by judging the values of these attributes with respect to {*T_j_*} according to Equations (10) and (11):

(10) $$y_{j}=〚x_{j}>T_{j}〛,1\leq j\leq 6;$$

(11) $$y_{j}=〚x_{j}<T_{j}〛,7\leq j\leq 8,$$

where 〚c〛 returns 1 when condition c exists or 0 otherwise.

Then the causes of perceptional degradation are analyzed from several dimensions, i.e., ISP, IP of CDN, segmented delay, time, TAC, phone model, and user. The main idea is to find the corresponding attributes of these dimensions whose ratio of disqualified samples (RDS) is lower than a predefined threshold.

Firstly, we analyze the cause of degradation from ISP point of view. The first packet RDS (i.e., *R_K_*), page RDS (i.e., *R_P_*), and service RDS (i.e., *R_S_*) of each ISP are calculated, according to following equations:

(12) $$R_{Ki}=\frac{\sum_{l=1}^{m}y_{1l}|x_{16l}=i}{m_{i}}\cdot 100\%$$

(13) $$R_{Pi}=\frac{\sum_{l=1}^{m}y_{2l}|x_{16l}=i}{m_{i}}\cdot 100\%$$

(14) $$R_{Si}=w_{k}R_{Ki}+\left(1-w_{k}\right)R_{Pi}$$

where *w_k_* is the weighting factor for *R_K_*. It depends upon the importance of *D_k_* to the overall service perception.

Then the overall service RDS (i.e., $\hat{R}$) is calculated:

(15) $$\hat{R}=\frac{1}{m}\overset{N}{\underset{i=1}{\sum }}R_{Si}\cdot m_{i}$$

For the ISP whose service RDS is larger than the threshold max($\hat{R}$, *T_s_*), it is then marked as “Disqualified ISP”. Here *T_s_* is a controlling threshold, to avoid false alarm in case all the ISPs are performing well.

The analysis of other dimensions can be carried out in the similar way as the ISP dimension, by defining corresponding RDS, including the segmented delay RDS (i.e., *R_SD_*), RSRP RDS (i.e., *R_RP_*), RSRQ RDS (i.e., *R_RQ_*), and coverage RDS (i.e., *R_C_*). In the analysis of TAC, phone model and user dimensions, only those with sufficient number of samples (i.e., larger than *T_m_*) are processed. We called them Major TAC, Major phone model, and Major User, respectively.

After all the dimensions are analyzed, we need to identify which causes are the most impacting ones. Here we define {${\tilde{R}}_{s}$}, the adjusted overall service RDS, for each identified cause of degradation. It refers to the overall service RDS by re-labelling all the disqualified samples resulting from this cause as qualified samples.

Finally, {${\bar{C}}_{v}\in \left(0,1\right],v=1\sim V$} is achieved, which means the normalized factors of significance (FoS) for each cause of perceptional degradation.

Algorithm 1 presents the pseudo code description of the proposed algorithm.

**Algorithm 1.** Analysis of Service Perception Degradation.**Input**: dataset $D=\left\{(x_{i},Y_{i})|1\leq i\leq m\right\}$,1. Labelling of disqualified samples with Equations (10) and (11);2. **for**
*i* = 1~*N*
**do**3.  calculate {*R_K_*},{*R_P_*},{*R_S_*} of the ISP with Equations (12)–(14);4. **endfor**5. calculate $\hat{R}$ with Equation (15);6. **for**
*i* = 1~*N*
**do**
7.  **if**
*R_Si_* > max($\hat{R}$, *T_s_*) **then**
8.   mark the website as a “Disqualified ISP”;9. **endfor**10. **for** every *Disqualified ISP*
**do**
11. **for** every *Major IP*
**do**
12.   calculate *R_S_* of the IP with Equations (12)–(14); 13.  **if**
*R_S_* > max($\hat{R}$, *T_s_*) **then**
14.   mark the IP as “Disqualified IP”; 15.  **endfor**16. **endfor**17. **for** every *segmented delay* {*D_dns_*, *D_tcp_*, *D_get_*, *D_res_*} **do**
18.  calculate *R_SD_* for each ISP with Equations (12)–(14); 19.  ${\hat{R}}_{sd}$ ← weighted mean of *R_SD_ w.r.t.* no. of samples; 20.   **if**
*R_SD_* > max(${\hat{R}}_{sd}$, *T_s_*) **then**
21.   mark the segmented delay as “Disqualified     Segmented Delay” of the ISP;22. **endfor**23. **for** every *hour* in the day **do**
24.  calculate *R_S_* of the hour with Equations (12)–(14); 25.  **if**
*R_S_* > max($\hat{R}$, *T_s_*) **then**
26.   mark the hour as “Disqualified Hour”;27. **endfor**
28. **for** every *Major TAC*
**do**
29.  calculate *R_S_* of the TAC with Equations (12)–(14); 30.  **if**
*R_S_* > max($\hat{R}$, *T_s_*) **then**
31.   calculate *R_RP_* and *R_RQ_* of the TAC;32.    *R_C_* ← $0.6R_{RP}+0.4R_{RQ}$; 33.   **if**
*R_C_* > ${\hat{R}}_{c}$
**then**
34.    mark the TAC as “Disqualified TAC”;35. **endfor**36. **for** every *Major Phone Model*
**do**
37.  calculate *R_S_* of the model with Equations (12)–(14); 38.  **if**
*R_S_* > max($\hat{R}$, *T_s_*) **then**
39.   mark the model as “Disqualified Terminal”;40. **endfor**41. **for** every *Major User*
**do**
42.  calculate *R_S_* of the user with Equations (12)–(14); 43.  **if**
*R_S_* > max($\hat{R}$, *T_s_*) **then**
44.   mark the model as “Disqualified User”;45. **endfor**46.  **for**
*v* = 1~*V*
**do**
47. calculate ${\tilde{R}}_{s}$ by re-labelling all the disqualified samples   resulting from the *v*-th cause of degradation as qualified ones; 48.  $C_{v}$ ← $R_{s}-{\tilde{R}}_{s}$;49. **endfor**50. ${\bar{C}}_{v}\in \left(0,1\right]$ ← normalization of $\left\{C_{v},\;v=1\sim V\right\}$;

### 4.2. Determination of Key Parameters

To guarantee their representativeness, the disqualification thresholds of the proposed algorithm should be defined based on dataset diversified enough in both spatial and temporal aspects. Thus the same dataset is employed as in Section 3.

The thresholds for {*D_k_*, *D_dns_*, *D_tcp_*, *D_get_*} are determined using the Q3+1.5IQR statistical method, which is widely used to find abnormal samples in a dataset. Note that HTTPS websites introduce more complicated interactions between terminals and the web server for authentication; therefore, the page delay of HTTPS websites is always larger than the page delay of HTTP websites. Therefore, the disqualification thresholds for HTTP and HTTPS websites are defined separately. Of the target websites used in this paper, Sina, Baidu, Weibo and Taobao use HTTPS, while the others use conventional HTTP.

**Figure 10:**
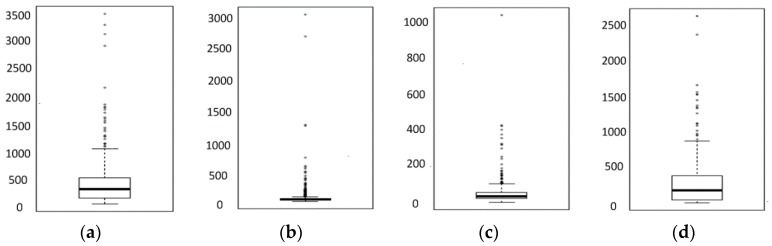
Boxplots of KQIs of HTTPS websites (unit: ms). (**a**) *D_k_*; (**b**) *D_dns_*; (**c**) *D_tcp_*; (**d**) *D_get_*.

Figure 10 presents a boxplot of {*D_k_*, *D_dns_*, *D_tcp_*, *D_get_*} of the HTTPS websites; the line of Q3+1.5IQR represents the disqualification thresholds. All the thresholds for both HTTPS and HTTP websites are illustrated in Table 4.

As seen in Figure 8, that page size has significant impact on the page delay for different ISP webpage. To mitigate such influence, the threshold of page delay for each ISP shall be defined by taking into consideration both the page size and page visit intensity. HTTP and HTTPS webpages shall also be considered separately.

Suppose the page size of the target HTTP websites in an area is {*P_i_*, *i* = 1~*n*}; the average page size is then $\bar{P}=\mathrm{mean}\left(P_{i}\right)$, and the percentage of page visit is {$\alpha_{i}$, *i* = 1~*n*} and $\underset{i}{\sum }\alpha_{i}=1$. We can then define the basic disqualification threshold of page delay as follows:

(16) $$T_{0}=\overset{n}{\underset{i=1}{\sum }}{\bar{D}}_{pi}\cdot \alpha_{i},$$

where ${\bar{D}}_{pi}$ is the Q3+1.5IQR of all the page delay samples of the *i*-th website which is achieved based on a long-term observation. The disqualification threshold of page delay for the *i*-th HTTP website can then be calculated by

(17) $$T_{i}=T_{0}\cdot \beta \cdot \frac{P_{i}}{\bar{P}},$$

where β is the controlling parameter and is determined with the relationship between page size and page delay observations. Thresholds for HTTPS websites can be determined in the same way. Here β is set to 1.1 and 0.54 for HTTP and HTTPS websites, respectively.

The disqualification thresholds for RSRP and RSRQ are −110 dBm and −10 dB, respectively; these are empirical values. Samples with RSRP and RSRQ lower than these thresholds are labelled as disqualified samples.

The default value of the remaining parameters is determined either from statistical or empirical point of view, as illustrated in Table 5 below.

## 5. Case Study and Discussion

In this section, using a CUP dataset acquired from the LTE network of Gansu province in August of 2017 as an example, we analyze the perceptional degradation of the province with the proposed algorithm. The time of data acquisition spans the whole month of August. Total volume of dataset is 711,714 contributed by 120,304 smartphone users in all the 14 cities of Gansu. As Gansu is a developing area in northwestern China, its telecom infrastructure is rather immature compared with eastern and southern China provinces.

**Figure 11:**
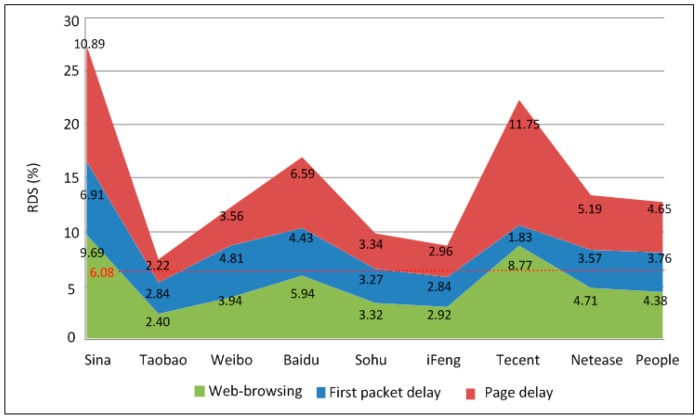
Output of Internet service provider (ISP) analysis. The red dotted line refers to the overall service ratio of disqualified samples (RDS).

Firstly, the output of the ISP analysis is presented in Figure 11, in which two websites, Sina and Tecent, are labelled with “Disqualified ISP” because their service RDSs exceed the overall service RDS $\hat{R}$ (which is 6.08% in this case).

Secondly, we further analyzed the disqualified ISPs from the IP point of view. IPs whose service RDS exceed $\hat{R}$ are marked as “Disqualified IP”. The results are given in Table 6.

It is seen that, the CDN nodes of all the four disqualified IPs locate in Guangdong province of south China, which is far from Gansu. Therefore, for developing regions like Gansu, the lack of local CDN deployment is a major cause of poor web browsing experience.

**Figure 12:**
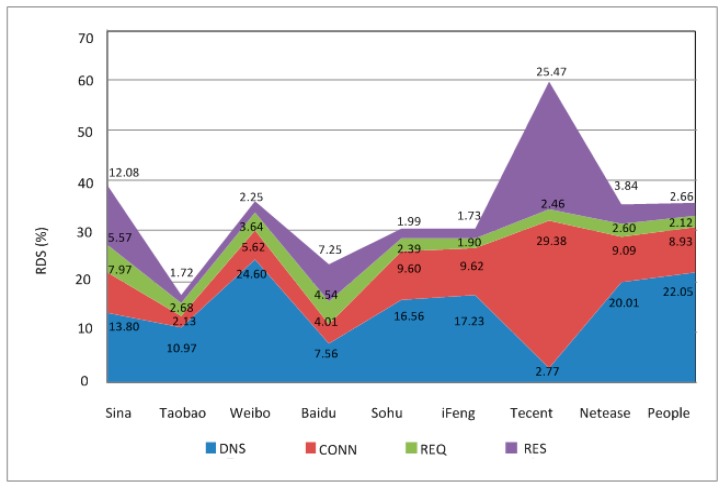
Output of segmented delay analysis.

The analysis of segmented delay is presented in Figure 12, where the $R_{sd}$ values of DNS delay, connection delay, request delay and response delay are 11.04%, 8.12%, 3.91% and 8.22%, respectively. Any corresponding RDS above those values is marked as a “Disqualified Segmented Delay”. In Figure 12, it is obvious that the DNS delay of most websites is disqualified. By further investigating the IP of the DNS server, it was found that most of the disqualified samples were from the IP 202.100.64.68; therefore, this IP should be checked carefully to improve the DNS delay performance.

**Figure 13:**
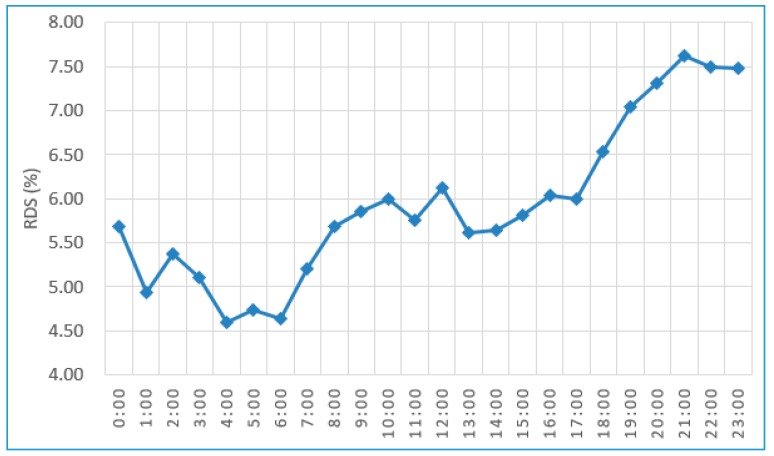
Temporal analysis.

The temporal analysis presented in Figure 13 shows that the RDS values for the time period from 18:00–24:00 are above the overall service RDS; consequently, these are marked with “Disqualified Time Period”.

From the coverage point of view, out of all the 152 TAC areas whose samples are above 1000, totally 41 TAC areas are marked with “Disqualified TAC”, because both the service RDS and coverage RDS values are higher than the thresholds. This means that poor signal quality might be the major reason for low service perception; thus, these TAC areas need further optimization.

**Figure 14:**
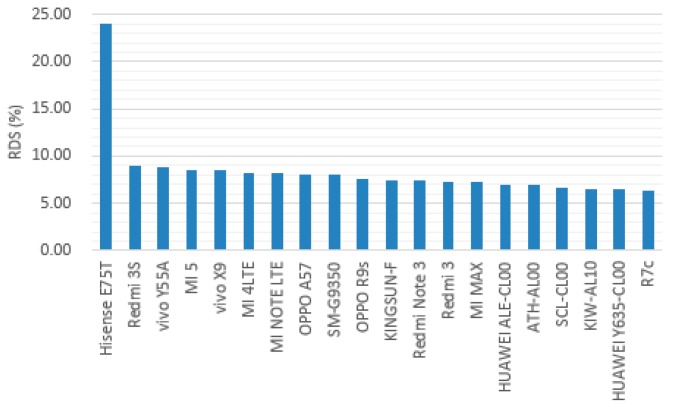
Terminal analysis.

There are a total of 440 models of smartphones in the dataset. Among which 74 models satisfies the minimal number of samples. Twenty models are marked as “Disqualified Terminal”, because their service RDSs exceed the threshold $\hat{R}$ (Figure 14). Among these, the service RDS of Hisense E75T is 24%, which is much higher than that of the others.

Let’s take a close look at Hisense E75T and try to find the reasons behind. Firstly, the diversity of all the 1562 samples is analyzed so as to exclude the accidental situations where most of the samples concentrate in a few days, hours, locations (i.e., cells), users or ISPs. Table 7 presents the calculation results.

It is seen that for all the dimensions, the max proportion among all the possible values is less than five times the average proportion. Thus we can say that the samples contributed by E75T are diversified enough. Having checked the hardware configurations of E75T we found that it is a low-end smartphone issued by May of 2016, with only 2 GB RAM and 1.3 GHz CPU employed. While the mainstream configurations in the same period are 3 GB RAM and 1.5 GHz CPU. Low hardware configuration might be the major reason for poor service perception.

As the distribution of samples among the users are extremely fragmented and there is no users with number of samples larger than *T_m_* in the dataset, it means the impact on service RDS from user point of view is marginal. Therefore, no user is marked as a “Disqualified User” in the user analysis.

**Figure 15:**
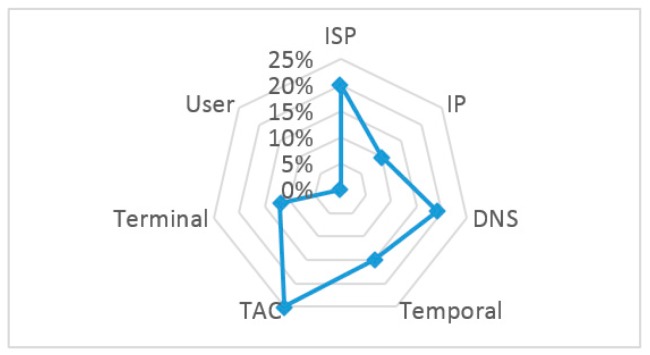
Radar map of the impact factors for overall service RDS.

Based on the marked disqualification factors mentioned above, the normalized factor of significance of all impact factors can then be achieved. To simplify our analysis, only the DNS delay is considered in the segmented delay. The results are illustrated in Figure 15 and Table 8.

As shown above, coverage, ISP and DNS are the top three impact factors for the overall service RDS of Gansu province during the time of measurement. Thus, these factors should be given high attention during daily network and service optimization to improve the overall user perception of web browsing services.

More specifically, more bandwidth and servers for CDN nodes 202.100.83.139 and 202.100.83.140 shall be added so as to take over the load of CDN nodes 183.61.38.230 and 14.215.138.13 far in Guangdong province. Similarly, local CDN nodes for website Sina shall be setup as well. For those TACs marked as “Disqualified TAC”, engineers should check carefully the cells within the TAC to further identify the base stations with low coverage and optimize them either by adjusting wireless parameters of the cell, or add more base stations where necessary. In addition, more bandwidth and servers shall be added for DNS server 202.100.64.68 to improve the DNS response delay and thus the first packet delay and page delay.

In summary, the proposed analytical framework and algorithm in this article can not only identify the actual impact factors that affect service perceptions in the target network but also identify the key factors that are in strong need of optimization. Thus, this framework provides a highly efficient way to support daily network maintenance and optimization efforts.

## 6. Discussion and Conclusions

In an age where OTT services are taking center stage, the large gap between network quality and user’s service perception has become the most annoying problem faced by conventional mobile network maintenance and optimization. Therefore, this paper presented a detailed analysis of which factors impact end-to-end OTT web browsing service perceptions and how these impacting factors affect service perceptions.

The analysis showed that the quality of wireless coverage is crucial to the first packet delay and page delay of web browsing. In weak coverage scenarios KQI deteriorates quickly. This clearly indicates that coverage is unambiguously an important factor for service perception. In addition, both the PCC and MIC correlation analyses showed that levels of service activity at different times of day are highly correlated with the KQI. The first packet delay and page delay are also highly correlated with each other.

On top of this, an analytical framework for the perceptional degradation of OTT web browsing service was proposed in this paper. The framework locates the actual impact factors of service perception degradation from seven dimensions: ISP, IP of the ISP or CDN nodes, segmented delays, time (as an alternative to network load), coverage, terminal, and user by utilizing the perceptional dataset crowdsensed from end-users’ smartphone. The contributions of these factors to the perceptional degradation, namely, the factors of significance, are also identified in the proposed algorithm. To our knowledge, this is a new attempt in this field and we found no similar works published so far.

Finally, the proposed algorithm was validated using a large perceptional dataset acquired through the CUP method from a live network in northwest China. The analysis results show that the method is able to capture the major impacting factors and the significance of their contributions to the overall service degradation. Specific suggestions of network adjustment can then be achieved to aid the engineers in their network maintenance and optimization work.

To our understanding, the proposed analytical framework is a statistical-based unsupervised learning, where all the entries of the input dataset are attributes, not labels in the sense of machine learning. All the possible reasons (e.g., if or not the disqualified ISP, IP, TAC, user, and terminal exist) behind the perception degradation are exactly the “labels” we are trying to find by the learning algorithm. For the next, we plan to explore semi-supervised learning in the reasoning of perception degradation, based on the dataset partially labelled by network maintenance engineers.

In addition, the best way of verifying whether the suggestions in the case study can really improve the service perception or not, since we had no cooperation with local network engineers of Gansu province. In the future, we are trying to have deep cooperation with operators on this.

A more effective method of perceptional degradation positioning should combine crowdsourced data with other sources of data, such as DPI and measurement report (MR) data and should take advantage of high-performance machine learning algorithms. These will form our major work directions in the future.

## Figures and Tables

**Table 1:** Ratio of Qualified Samples (RQS) index tree.

Level of Indices	Type	Index Name
4	Overall	Overall RQS
3	Network-level	3G RQS, 4G RQS
2	Service-level	Web-browsing service RQS, Video service RQS
1	KQI-level	Fist packet delay RQS, Page delay RQS, Video stalling RQS, Video download rate RQS

**Table 2:** Comparison of the traditional and OTT webpage.

	Sohu (Traditional)	Sohu (OTT)	Tecent (Traditional)	Tecent (OTT)
Home page URL	www.sohu.com	m.sohu.com	www.qq.com	portal.3g.qq.com
Size of frame	179 KB	72 KB	228 KB	123 KB
Size of whole page	1.58 MB	1.14 MB	1.69 MB	383 KB
No. of resources	49	13	86	8
Downloading and rendering strategy	Full page	On-demand	Full page	On-demand

**Table 3:** Comparison of impact factors of service perception.

Factor	Significance	Range of Influence	Reasons Behind
Access nwk	Large	Medium	Propagation environment, signal strength & quality
Core nwk	Small	Large	Malfunction of nodes & links
Terminal	Small	Medium to Large	HW configuration & SW capability (incl. OS)
User	Small	Small	Individual difference of user habits and psychological anticipation of QoE
ISP	Large	Medium to Large	Load, processing capability & bandwidth of CDN nodes
Temporal	Medium	Medium	Temporal difference of service demand and network load

**Table 4:** Disqualification Thresholds of KQIs for both HTTP and HTTPS Websites.

KQI	Threshold (HTTP) (ms)	Threshold (HTTPS) (ms)
*D_k_*	726	1049
*D_dns_*	138	68
*D_tcp_*	79	150
*D_get_*	446	865

**Table 5:** Setting of other parameters.

Parameter	*T_m_*	${\hat{R}}_{c}$	*w_k_*	*T_s_*
Value	1000	20%	0.3	5%

**Table 6:** IP analysis of disqualified ISP.

ISP	IP of CDN Nodes	Portion of Samples	Service RDS	Disqualified IP	IP Service Provider
Tecent	202.100.83.140	35%	2.65%	No	Gansu Telecom
	202.100.83.139	31%	2.32%	No	Gansu Telecom
	183.61.38.230	13%	**17.98%**	**Yes**	Guangdong Telecom
	14.215.138.13	13%	**18.66%**	**Yes**	Guangdong Telecom
Sina	183.60.93.249	71%	**6.96%**	**Yes**	Guangdong Telecom
	14.215.135.31	26%	**16.89%**	**Yes**	Guangdong Telecom

**Table 7:** Diversity analysis of Hisense E75T data samples.

	IMSI	Date	Hour	cellID	ISP
No. of values	150	31	24	243	9
Avg. proportion for each value	0.7%	3.2%	4.2%	0.4%	11.1%
Max proportion	2.9%	11%	11.8%	1.7%	12.8%
Max/Avg. proportion	4.4	3.4	2.8	4.1	1.2

**Table 8:** IP analysis of disqualified ISP.

Factors to be Solved	Gain in Overall Service RDS (%)	Normalized FoS
ISP	2.44	**20%**
IP	1.20	10%
DNS	2.38	**19%**
Temporal	1.85	15%
TAC	3.06	**25%**
Terminal	1.43	12%
User	0.00	0%

## Acronyms

OTT	over-the-top
MNO	mobile network optimization
DT	drive test
CQT	call quality test
KPI	key performance indicator
QoE	quality of experience
KQI	key quality indicator
QoS	quality of service
DPI	deep packet inspection
CUP	crowdsourcing-based user perception
LTE	long term evolution
NPS	net promotion score
MCS	mobile crowdsensing
APP	application
SDK	software development kit
OS	operating system
UE	user equipment
ISP	Internet service provider
RAN	radio access network
CN	core network
IMEI	international mobile equipment identity
IMSI	international mobile subscriber identity
HTTP	hypertext transfer protocol
DNS	domain name system
TCP	transmission control protocol
RSRP	reference signal received power
RSRQ	reference signal received quality
TAC	tracking area code
RQS	ratio of qualified samples
CDN	content distribution network
PCC	Pearson correlation coefficient
MIC	maximum information coefficient
RDS	ratio of disqualified samples
MR	measurement report
